# Long-term persistence of treatment after hip fracture in a fracture liaison service

**DOI:** 10.1038/s41598-022-13465-x

**Published:** 2022-06-07

**Authors:** Antonio Naranjo, Amparo Molina, Adrián Quevedo, Francisco J. Rubiño, Fernando Sánchez-Alonso, Carlos Rodríguez-Lozano, Soledad Ojeda

**Affiliations:** 1 Rheumatology Department, Hospital Universitario de Gran Canaria Dr. Negrín, Barranco de La Ballena, 35011 Las Palmas, Spain; 2 University of Las Palmas de Gran Canaria, Las Palmas, Spain; 3 Investigation Unit, Spanish Society of Rheumatology, Madrid, Spain

**Keywords:** Rheumatology, Metabolic disorders, Rheumatic diseases, Trauma

## Abstract

Long-term adherence to antiosteoporosis medication (AOM) in the setting of a fracture liaison service (FLS) are not well known. Patients ≥ 50 with hip fracture seen in an FLS and recommended for treatment to prevent new fractures were analyzed. Baseline data included demographics, identification mode, previous treatment and FRAX items. Patient records were reviewed 3–8 years later, and these data were collected: (1) survival; (2) major refracture; (3) initiation of treatment, proportion of days covered (PDC) and persistence with AOM. 372 patients (mean age, 79 years; 76% women) were included. Mean follow-up was 47 months, 52 patients (14%) had a refracture (22 hip) and 129 (34.5%) died. AOM was started in 283 patients (76.0%). Factors associated with initiation of AOM were previous use of bisphosphonate (OR 9.94; 95% CI 1.29–76.32) and a lower T-score lumbar (OR 0.80; 95% CI 0.65–0.99). Persistence decreased to 72.6%, 60% and 47% at 12, 36 and 60 months. A PDC > 80% was confirmed in 208 patients (55.7%) and associated with previous use of bisphosphonate (OR 3.38; 95% CI 1.34–8.53), treatment with denosumab (OR 2.69; 95% CI:1.37–5.27), and inpatient identification (OR 2.26; 95% CI 1.18–4.34). Long-term persistence with AOM was optimal in patients with hip fracture seen at an FLS. A PDC > 80% was associated with inpatient identification and prescription of denosumab.

## Introduction

Hip fracture is a common, serious, and costly injury that must be managed with surgery. It is associated with both residual disability and high mortality and is much easier to identify and register than other osteoporotic fractures. The population incidence of hip fracture is increasing worldwide due to rising life expectancy. Antiosteoporosis medication (AOM) for secondary prevention of osteoporotic fracture is prescribed infrequently in many countries, despite the recommendations of medical societies. The adherence to AOM reported as proportion of days covered (PDC) at 1 year after hip fracture in standard care is as low as 6–13%^1^.

The fracture liaison service (FLS) model is the standard of care for patients with a fragility fracture because of its effectiveness in terms of initiation of and adherence to treatment, refractures, and survival^2^. Demonstrating this effectiveness is key when promoting the FLS model in various countries and regions and when attempting to ensure funding by managers. Given that reducing the number of new fractures and increasing survival (main objectives) involve a high of cases and many years of follow-up at regional level, the surrogate indicator of initiation of and adherence to treatment is more feasible.

PDC is undoubtedly a more demanding indicator than adherence. PDC or adherence > 80% has been associated with more effective treatment in terms of reducing the frequency of fracture^3^.

Also essential for the care of the patient with hip fracture in an FLS is the means used to identify and follow the patient, namely, during admission or through an outpatient visit a few weeks after discharge. Some FLS models serve only outpatients, others serve only inpatients, and some models are mixed. The objectives of our study were to analyze in a mixed FLS model: (1) the long-term persistence of treatment with AOM and PDC after hip fracture and associated factors; and (2) the risk of major osteoporotic refracture.

## Methods

We designed an observational, longitudinal, prospective, and retrospective study to analyze 5-year persistence of treatment and associated factors after hip fracture at an FLS. The structure and operation of our mixed FLS have been addressed elsewhere^4^. The University hospital Dr. Negrín (Gran Canaria, Spain) provides health coverage to approximately 400,000 inhabitants from 22 primary care health centers linked to the hospital.

### Patients

The study population comprised patients older than 50 years with a fragility hip fracture managed in the FLS between 2012 and 2018. Patients with pathologic fractures, severe functional disability, very short life expectancy, advanced liver or renal disease, or with any other severe condition that would render patients unable to participate in the program were excluded. Our FLS actively identifies patients with hip fracture at 3 points in the care process: (a) inpatient setting with an trained FLS nurse; (b) outpatients captured through emergency registries and attended by a trained FLS nurse; and (c) outpatient rheumatology clinic, where staff physicians attend patients with fracture referred by other specialties and primary care.

#### Prospective study (baseline visit)

A medical history was taken in all cases, laboratory tests and densitometry bone densitometry (DXA) were performed (DXA was not performed in those admitted), and treatment was recommended. Treatment was prescribed by either primary care physicians or rheumatologists. Most patients were assessed by a trained nurse who referred the patient to a primary care physician for initiation of a specific treatment. The nurse followed a study protocol and was supervised by a medical coordinator. The data collected at the baseline visit were as follows: demographic data, previous treatment, previous fracture, family history of hip fracture in either parent, tobacco or alcohol consumption, treatment with corticosteroids, history of rheumatoid arthritis, and secondary osteoporosis. The type of hip fracture was also recorded (pertrochanteric, intracapsular, or subtrochanteric).

During this first visit, a DXA scan and laboratory tests were also performed. The risk of fracture was calculated using the FRAX tool for the Spanish population (https://www.sheffield.ac.uk/FRAX/tool.aspx?lang=sp). Dichotomous variables were adjusted for weight and height, and the T score was recorded at the femoral neck.

Finally, general measures (diet, exercise, advice on preventing falls) and treatment with calcium, vitamin D, and an AOM (bisphosphonate, denosumab, or teriparatide) were recommended to all patients, unless they had severe kidney disease or very low life expectancy (exclusion criteria). Patients seen during hospitalization for hip fractures followed a similar protocol except for DXA. Patients whose treatment had failed (incident fracture in patients already under treatment for > 12 months), patients with a contraindication to oral bisphosphonates/denosumab, and patients with multiple fractures who were candidates for treatment with zoledronate or teriparatide were referred to the rheumatology osteoporosis clinic. We included patients who had been recommended treatment with AOM. Thus, in this study 100% of the patients were recommended AOM. Short-term follow-up was carried-out by the FLS, mostly through telephone calls and a review of the electronic prescription at 3, 6, and 12 months.

#### Retrospective study (long-term follow-up)

The clinical history of the patients was reviewed in the period between June 2019 and October 2020. The following variables were obtained from the records: (1) cause and date of death (if applicable); (2) new confirmed incident fractures (hip, vertebra, humerusand forearm) reviewing the radiographs available on the health area platform and all reports from the emergency department. In the case of patients with more than one incident fracture, the most important was considered following this order: hip, vertebra, humerus and forearm; (3) initiation of treatment and adherence of AOM. Persistence was defined as withdrawal of AOM from the pharmacy, that is continuing the treatment for the prescribed duration (computer platform). The persistence of AOM is the verification that the patients effectively continue the prescribed treatment and is expressed as the percentage of patients with respect to the total number of patients alive on each control date. Deceased patients were excluded from the formula at each control time of persistence.

PDC is calculated based on the number of days supply a drug is dispensed for, divided by the number of days the prescription is in the patient's possession. The PDC, therefore, is expressed as the percentage of time that the patient has performed the treatment in a given period of time. In Spain, drugs are covered for the most part by the public health system; in particular, patients aged ≥ 65 years do not pay for medicines or pay only a nominal amount. Patients with insufficient data in their electronic pharmacy record were excluded.

#### Standard of care

We selected a group of patients attended in our hospital for hip fracture in the first quarter of 2015 in whom FLS activity stopped owing to sick leave of the nurse. The purpose of the control group was only to have a reference of the real practice in our health area when the activity of the FLS unit stops. These patients followed standard care after hospital discharge. This group was only analyzed for adherence to AOM, which was obtained through the electronic records in the same way as patients managed in the FLS.

#### Statistical analysis

A descriptive statistical analysis was performed. Differences between groups (initiation vs no initiation of treatment, adherent patient vs non-adherent and refractured vs non-refractured) were assessed using the *t* test in the case of continuous variables and the chi-square and Fisher exact tests in the case of categorical variables. Logistic regression models were run when more than 1 variable was found to be associated with prescribed AOM and proportion of days covered (PDC) with antiosteoporosis medication higher and lower than 80% to build up the model, we started including variables that had a p-value lower than 0.2 and other suggested by their clinical relevance. Model were build adding variables until we decide a model who has collinearity problem and think is still useful as explanatory model.. Survival was estimated using Kaplan–Meier analysis, and hazard ratios (HR) were estimated using Cox regression analysis. Data were analyzed using Stata version 13.1. A p-value less than 0.05 was considered statistically significant.

### Ethics approval and consent to participate

The institutional review board of our hospital (CEIC Hospital Universitario de Gran Canaria Dr. Negrin; code NAR-OST-2011-02) approved the program protocol and the study. All participants provided written informed consent for their data to be used for research, including informed consent obtained from the next of kin or legal guardian for dead patients. All methods were carried out in accordance with guidelines and regulations of “Declaration of Helsinki”.

## Results

### Baseline characteristics

**Figure 1:**
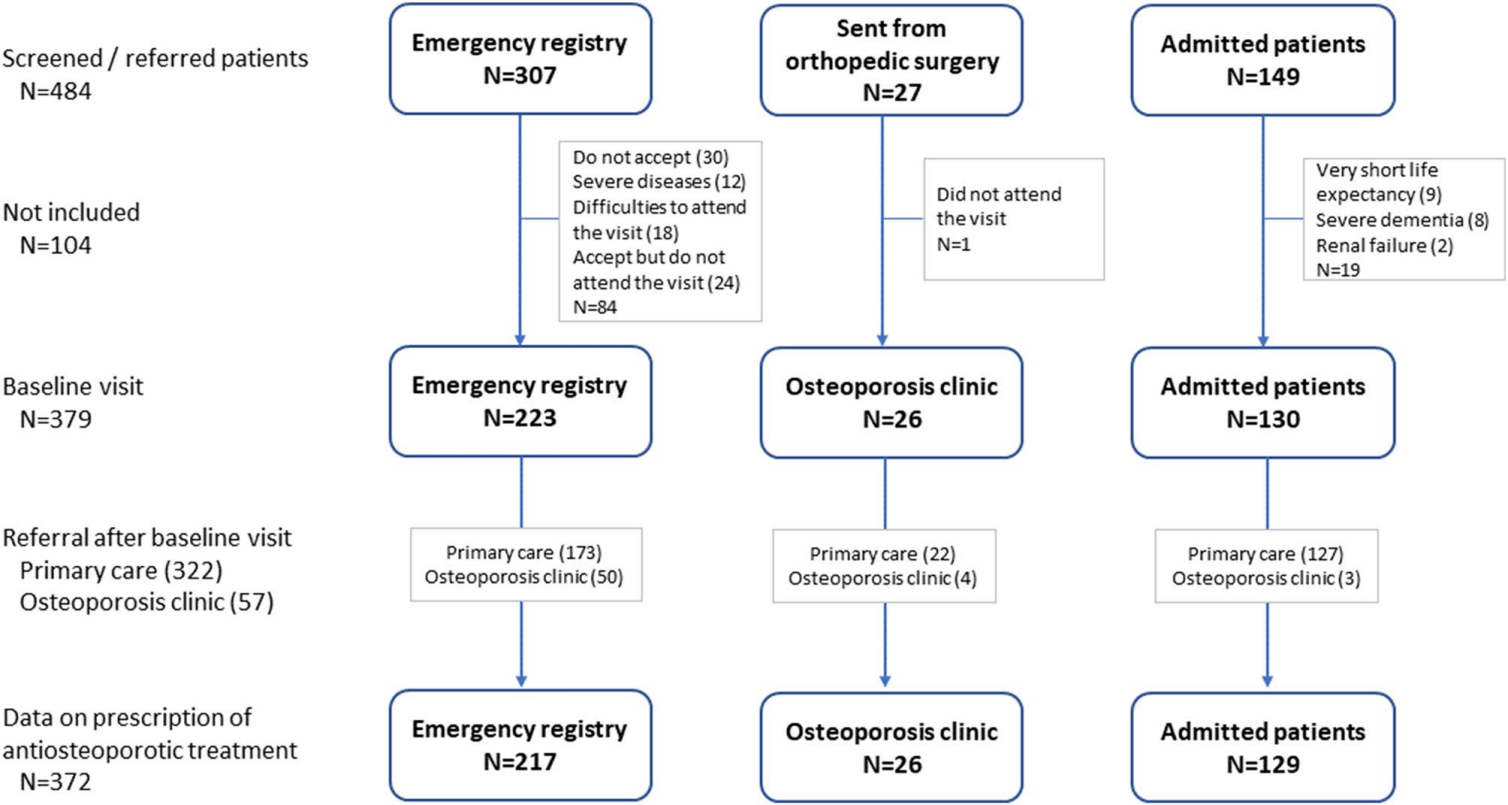
Flow diagram of patients. Edited with Microsoft 365 A3 for faculty.

A total of 484 patients were screened (Fig. 1), 379 of whom attended the baseline visit and 7 were excluded because of insufficient data on prescription of AOM. Thus, we analyzed 372 patients (mean age, 79 years; 76% women) (Table 1). Patients were most often identified via the emergency registry (58% of cases) and attended as outpatients several weeks after the fracture. Patients identified during admission were older than outpatients (83.9 vs 77.7 years; p < 0.001). The mean delay between the date of fracture and baseline visit to the FLS was 17 weeks (SD 14, range 0–52), with a delay of under 12 weeks in 145 patients (39%). Pertrochanteric fracture was the most frequent type (51%).

**Table 1:** Baseline characteristics of patients.

	Patients (N = 372)
Age (years) mean (SD), range	79.4 (8.9), 53–102
Sex (female), n (%)	283 (76.1)
**Type of fracture, n (%)**	
Pertrochanteric	191 (51.3)
Intracapsular	151 (40.6)
Subtrochanteric	24 (6.4)
Others	6 (1.6)
**Identification of patients, n (%)**	
Emergency registry	217 (58.3)
Admission	129 (34.6)
Rheumatology osteoporosis clinic	26 (6.9)
**Previous treatment with bisphosphonates, n (%)**^a^	53 (14.4)
**Risk factors for fracture, n (%)**	
Previous fragility fracture	76 (20.4)
Parental hip fracture	35 (9.4)
Smoking	37 (9.9)
Corticosteroids	40 (10.7)
Rheumatoid arthritis	10 (2.7)
Secondary osteoporosis	59 (15.8)
Alcohol	17 (4.6)
**Densitometry results, n (%)**^**a**^	
Normal	11 (5.3)
Osteopenia	81 (39.1)
Osteoporosis	115 (55.5)
T score lumbar spine, mean (SD)	− 1.9 (1.6)
T score femoral neck, mean (SD)	− 2.4 (1.0)
**FRAX**	
FRAX major, mean (SD)	18.4 (10.2)
FRAX hip, mean (SD)	9.7 (8.2)
**Referral after baseline visit, n (%)**^**a**^	
Rheumatology osteoporosis clinic	57 (15.3)
Primary care	315 (84.7)

^a^Previous treatment recorded in 369 patients. Densitometry values were recorded in 207 patients.

Fourteen percent of patients had previously undergone treatment with AOM in the form of bisphosphonates, and the most frequent risk factors for fracture were previous fracture and secondary osteoporosis. Fifty-five percent of the DXA studies revealed osteoporosis, and 84% of patients were referred to their primary care center for prescription of treatment (Table 1).

### Follow-up

The mean follow-up was 46.9 months (SD 22, range 0.5–84 months). The minimum follow-up in survivors was 37 months.

A total of 129 patients died (34.5%; 42% male [37/89] and 32% female [92/283]; p = 0.068). Of these, 49 (13.1%) died in the 12 months after their baseline visit and 23 (6.1%) died during the second year.

Refracture occurred in 52 patients (13.9%; 15% female and 9% male; p = 0.12), 22 of whom (5.9%) were hip refractures.

### Treatment initiation

AOM was initiated during the first 3 months in 283 patients (76.0%); this consisted of a bisphosphonate in 183 patients (64.6%; alendronate in 145, risedronate in 34, ibandronate in 4) and denosumab in 100 patients (35.3%). No patients were prescribed teriparatide or zoledronate.

Table 2 presents the comparison between patients who were prescribed and initiated AOM after hip fracture and those for whom no medication was initiated. The bivariate analysis revealed an association between initiation of treatment and previous treatment with a bisphosphonate, female sex, previous fracture, and FRAX major. However, we did not find an association with the type of hip fracture, other risk factors for osteoporosis and fracture, delayed care in FLS after the fracture, referral after the visit, or DXA results.

**Table 2:** Comparison of patients who were prescribed antiosteoporosis medication and those who were not.

	No prescription	Prescription at any time	OR (95% CI)	p	Model OR (95% CI)	p
	(n = 89)	(n = 283)				
Age (years), mean (SD)	81.0 (8.5)	78.9 (9.0)	0.97 (0.95–1.00)	0.054	0.94 (0.90–0.99)	0.001
Sex (female), n (%)	55 (61.8)	228 (80.5)	2.56 (1.52–4.31)	0.0001	1.72 (0.79–3.74)	0.168
Identification of patients, n (%)						
Emergency registry	54 (60.6)	163 (57.6)	0.55 (0.18–1.66)	0.289		
Admission (hip fracture)	31 (34.8)	98 (34.6)	0.57 (0.18–1.80)	0.341		
Attended < 12 weeks after the fracture	32 (35.9)	113 (39.9)	1.16 (0.71–1.91)	0.55		
Previous fracture, n (%)	10 (11.2)	67 (23.6)	2.45 (1.20–5.00)	0.014		
Previous treatment with bisphosphonates, n (%)^a^	2 (2.2)	51 (18.0)	9.58 (2.28–40.19)	0.002	9.94 (1.29–76.32)	0.027
Lumbar T score, mean (SD)	− 1.38 (1.6)	− 2.05 (1.6)	0.78 (0.64–0.95)	0.014	0.80 (0.65–0.99)	0.039
Densitometry result; osteoporosis, n (%)^a^	22 (24.7)	92 (32.5)	1.89 (1.00–3.59)	0.05		
FRAX major, mean (SD)	19.0 (10.2)	16.4 (9.9)	1.03 (1.00–1.05)	0.04		
Referral to primary care after baseline visit, n (%)	79 (88.7)	237 (83.7)	0.64 (0.31–1.32)	0.22		

^a^Previous treatment recorded in 369 patients. Densitometry values were recorded in 207 patients.

In the multivariate model (including age, sex, previous treatment with AOM, previous fracture, lumbar T-score, and FRAX), the factors associated with initiation of treatment were a younger age (OR 0.94; 95% CI 0.90–0.99), previous treatment with a bisphosphonate (OR 9.94; 95% CI 1.29–76.32), and a lower lumbar T-score (OR 0.80; 95% CI 0.65–0.99) (Table 2).

### Treatment adherence

A PDC > 80% was confirmed in 208 patients (55.7%) (Table 3), 73.4% of whom started AOM (63% of those attended the outpatient osteoporosis clinic at some point, and 54% who were referred to their general practitioner; p = 0.23). In the bivariate analysis, the factors associated with PDC > 80% were female sex, initial visit during admission, previous fracture, previous bisphosphonates, prescription of denosumab, and FRAX major. However, the type of fracture, the delay in visit to the FLS, the lumbar T-score, and referral after the visit were not associated with PDC > 80%. In the multivariate model (including sex, previous treatment with AOM, previous fracture, initial visit during admission, FRAX major, and prescription of denosumab), a PDC > 80% was associated with previous treatment with a bisphosphonate (OR 3.38; 95% CI 1.34–8.54), prescription of denosumab versus an oral bisphosphonate (OR 2.70; 95% CI 1.38–5.27), and inpatient identification (OR 2.26; 95% CI 1.18–4.34).

**Table 3:** Comparison of patients with proportion of days covered (PDC) with antiosteoporosis medication higher and lower than 80%.

	PDC < 80	PDC > 80	OR (95% CI)	p	Model OR (95% CI)	p
	(n = 164)	(n = 208)				
Age (years), mean (SD)	79.1 (9.5)	79.5 (8.4)	1.01 (0.98–1.03)	0.52		
Sex (female), n (%)	110 (67.0)	173 (83.1)	2.43 (1.49–3.95)	0.0001	1.12 (0.62–2.23)	0.568
Identification of patients (ref emergency registry), n (%)						
Admission (hip fracture)	47 (28.6)	82 (39.4)	1.76 (1.13–2.75)		2.26 (1.18–4.34)	0.014
Previous fracture, n (%)	26 (15.8)	51 (24.5)	1.72 (1.02–2.91)	0.04		
Previous treatment with bisphosphonates, n (%)^a^	8 (4.8)	45 (21.6)	5.38 (2.46–11.78)	0.0001	3.38 (1.34–8.54)	0.01
Densitometry result; osteoporosis, n (%)^a^	55 (33.5)	59 (28.3)	1.30 (0.75–2.26)	0.28		
Lumbar T score mean (SD)	− 1.856 (1.701)	− 1.933 (1.603)	0.97 (0.82–1.15)	0.74		
FRAX major, mean (SD)	16.4 (10.2)	19.9 (10.0)	1.04 (1.01–1.06)	0.001		
Referral to primary care after baseline visit, n (%)^a^	143 (87.1)	172 (82.6)	0.70 (0.39–1.26)	0.23		
Start denosumab vs bisphosphonates	14 (8.5)	86 (41.3)	3.07 (1.61–5.84)	0.0001	2.70 (1.38–5.27)	0.004

^a^ Previous treatment recorded in 369 patients. Densitometry values were recorded in 207 patients.

**Figure 2:**
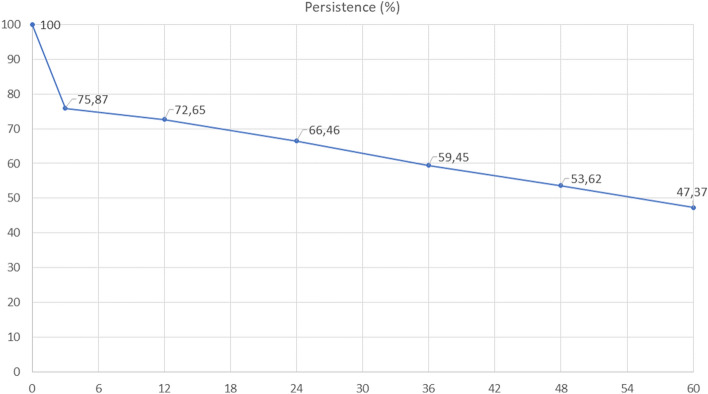
Persistence of treatment with antiosteoporosis medication over the 5-year follow-up period. 100% of patients were recommended antiosteoporotic treatment. Edited with Microsoft 365 A3 for faculty.

Figure 2 shows the persistence of AOM over the 5-year follow-up period. Persistence decreased to 72.6% at 12 months, 59.4% at 36 months, and 47% at 60 months. Of the 283 patients who started AOM, 36 required their treatment to be changed; (from a bisphosphonate to denosumab in 31 cases, to a different bisphosphonate in 4 cases, and from denosumab to a bisphosphonate in 1 case).

We analyzed 89 consecutive patients who did not attend the FLS in the first quarter of 2015 (they were not invited due to the nurse's sick leave). The mean age was 80.7 years, and 73% were women. At 3, 12, 24, and 36 months after discharge, 21.3%, 14.2%, 13.1%, and 12.5%, respectively, were receiving AOM.

**Figure 3:**
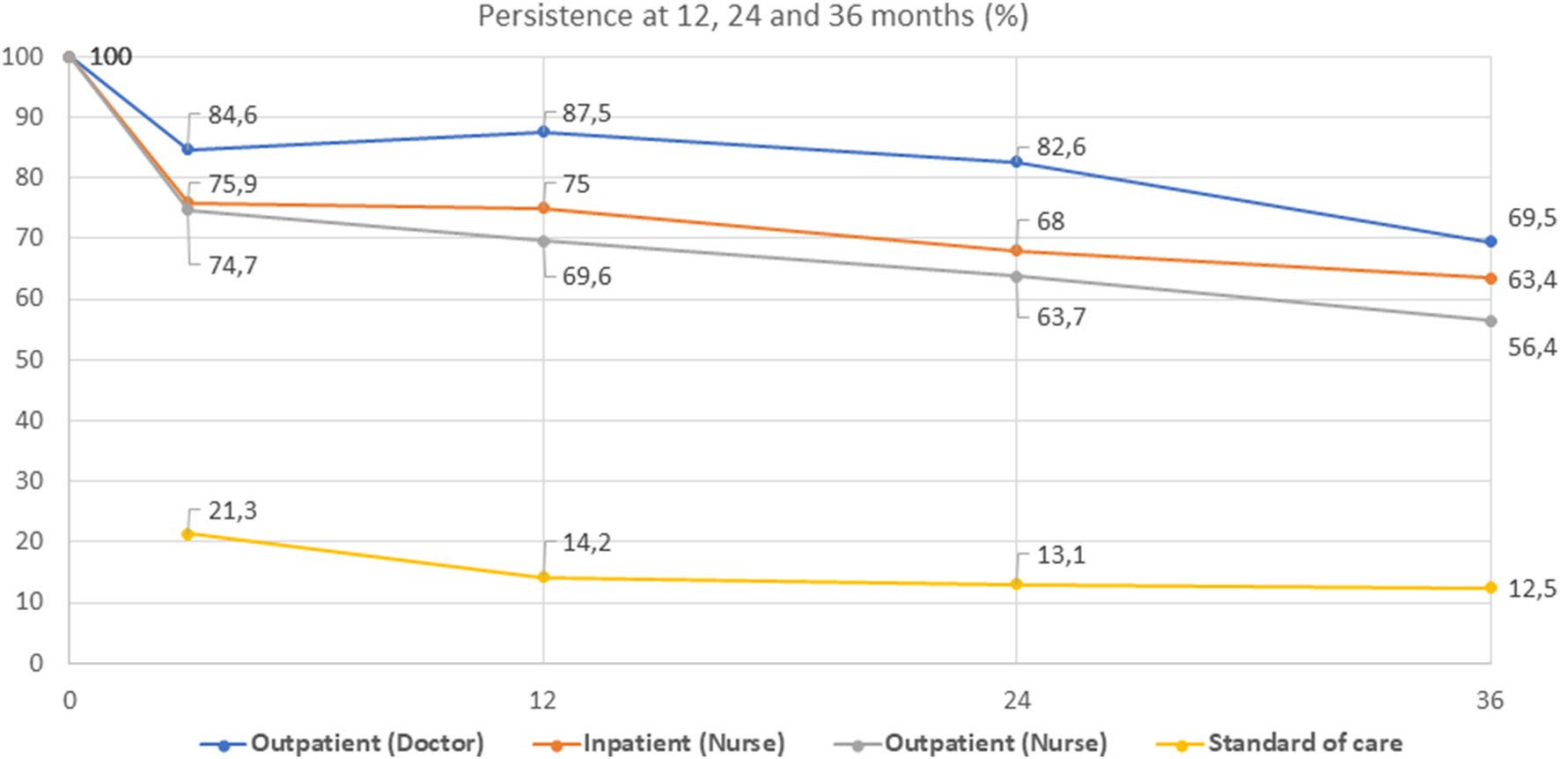
Persistence of treatment with antiosteoporosis medication over a 3-year follow-up in patients and controls. 100% of patients from FLS were recommended antiosteoporotic treatment. Edited with Microsoft 365 A3 for faculty.

Figure 3 shows the persistence of AOM over a 3-year follow-up period comparing inpatient FLS, outpatient FLS-nurse, outpatient/osteoporosis clinic, and standard care.

**Figure 4:**
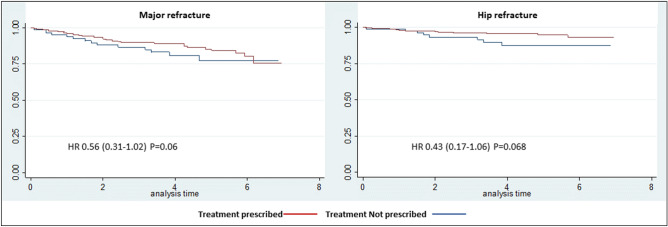
Kaplan–Meier estimates for refracture over 7 years of follow-up.

Major refracture occurred in 13.4% of patients with a prescription and in 15.7% of those without. Hip refracture occurred in 4.6% of patients with a prescription and in 8.9% of those without a prescription Table 4 shows the baseline characteristics of patients with and without refracture during follow-up. A Kaplan–Meier analysis taking into account the months that each patient remained with treatment, revealed that re-fracture free probability in patients who started AOM was higher than in those patients not prescribed AOM. We estimated crude HR and HR adjusted by age, sex and previous treatment. In both cases, the prescription of AOM was associated with a lower risk of refracture. HR for major fracture and hip fracture were 0.59 (0.33–1.05) and 0.46 (0.19–1.11) and adjusted were HR 0.56 (0.31–1.02) P = 0.06 and 0.43 (0.17–1.06) P = 0.068 respectively (Fig. 4).

**Table 4:** Comparison of baseline characteristics of patients with and without refracture during follow-up.

	No refracture (N = 320)	Refracture (N = 52)	P-value
Age (years) mean (SD), range	79.8 (8.7)	77.9 (10.0)	0.1659
Sex (female), n (%)	239 (74.7)	44 (84.6)	0.12
**Type of fracture, n (%)**			
Pertrochanteric	169 (52.8)	22 (42.3)	0.577
Intracapsular	126 (39.4)	25 (48.1)	
Subtrochanteric	20 (6.3)	4 (7.7)	
Others	5 (1.6)	1 (1.9)	
**Identification of patients, n (%)**			
Emergency registry	184 (57.5)	33 (63.5)	0.383
Admission	115 (35.9)	14 (26.9)	
Rheumatology osteoporosis clinic	21 (6.6)	5 (9.6)	
Previous treatment with bisphosphonates, n (%)^a^	45 (14.2)	8 (15.7)	0.778
**Risk factors for fracture, n (%)**			
Previous fragility fracture	63 (19.7)	14 (26.9)	0.232
Parental hip fracture	29 (9.1)	6 (11.5)	0.576
Smoking	29 (9.1)	8 (15.4)	0.158
Corticosteroids	35 (11.0)	5 (9.6)	0.77
Rheumatoid arthritis	9 (2.8)	1 (2.0)	0.723
Secondary osteoporosis	49 (15.3)	11 (21.2)	0.288
Alcohol	14 (4.4)	3 (5.8)	0.655
**Densitometry results, n (%)**^**a**^			
Normal	10 (5.8)	2 (5.9)	0.879
Osteopenia	69 (39.9)	12 (35.3)	
Osteoporosis	94 (54.3)	20 (58.8)	
T score lumbar spine, mean (SD)	− 1.8 (1.6)	− 2.4 (1.6)	0.073
T score femoral neck, mean (SD)	− 2.4 (1.1)	− 2.8 (1.0)	0.088
**FRAX**^**®**^			
FRAX^®^ major, mean (SD)	18.2 (10.1)	19.8 (10.9)	0.304
FRAX^®^ hip, mean (SD)	9.6 (8.1)	10.9 (9.5)	0.284
**Referral after baseline visit, n (%)**^**a**^			
Rheumatology osteoporosis clinic	50 (15.6)	7 (13.5)	0.688
Primary care	270 (84.4)	45 (86.5)	

^a^Previous treatment recorded in 369 patients. Densitometry values were recorded in 207 patients.

## Discussion

The present study reports on long-term persistence of treatment with AOM in patients with hip fracture in the context of FLS. Our comparison of FLS with standard management clearly shows the differences in persistence of treatment after hip fracture (72% vs 14% at 12 months and 59% vs 12% at 3 years). In addition, our data support the effectiveness of the FLS model for hip fracture, and indicate that recommendations on treatment should be made at admission, since the persistence to treatment is higher and the number of patients included might be potentially higher, and that the approach could prove more cost-effective, given the lower number of outpatient visits and tests such as DXA.

Only a fifth of patients who sustain a fragility fracture receive AOM^5^. Analysis of the studies included in a systematic review showed lower mortality after hip fracture associated with AOM, mostly bisphosphonates^6^. Kim et al. reported the use of AOM in 3 cohorts of patients after hip fracture (United States, Korea, and Spain). In the year before the index fracture, 16–18% were taking AOM, with an increase to 11–39% at 3 months. For those who received ≥ 1 prescription for AOM, the mean proportion of days covered in the year after the fracture was 43–70%^1^. Another study of hip fracture found that 48% of patients started AOM after discharge, but that only 34% of those prescribed treatment reported still taking their medication 1 year later^7^. In other words, only 16.3% of the original total were on treatment 12 months after discharge. A multicenter, prospective cohort of 5456 hip fracture patients in England and Wales found that 52% were prescribed AOM at discharge and, of these, 33% reported still taking their medication 120 days later^8^, that is, 17% of the original total were taking AOM 4 months after discharge. These results are in line with our data on standard care: persistence in patients not cared for by the FLS in our hospital was 14% at 12 months.

In its 2019 summary (13,181 patients), the Spanish registry of hip fracture reported the prescription of AOM in 5.9% of patients before the fracture and 42.8% at 30 days after discharge. These data were similar to those reported in the 2017 and 2018 summaries^9^. In the analysis of UK primary care prescriptions of any AOM after hip fracture, 5% of patients were prescribed AOM in 1999, increasing to 51% in 2011 before decreasing to 39% in 2013^10^. Neither the Spanish registry nor the UK registry^9,10^ has reported 1-year adherence, which, as previously described, is usually reduced by approximately half in standard care.

In summary, initiation of AOM after hip fracture in standard care seems to have improved in recent years in some countries, although adherence at 12 months remains low (around 20%).

The FLS model is associated with more frequent initiation of treatment. One systematic review found that, compared with standard management, FLS-based care increased initiation of treatment by 20 percentage points^2^. However, adherence rates vary depending on the study. For example, a US study found that of the 1840 patients from an FLS who were initially prescribed medication, 77% initiated AOM and 62% remained adherent to treatment^11^. Chandran et al.^12^ reported a 2-year adherence of 75% in 938 patients from Singapore, while a study of patients discharged from an FLS ≥ 12 months in Australia showed that 74% self-reported adherence to bisphosphonates^13^. In an FLS in Lille, France, parenteral drugs were prescribed to most patients, with a primary adherence of 75%^14^. However, results from other FLS experiences were less optimistic. The FLS Database of the Royal College of Physicians reported a mean adherence at 12 months of 23% (range, 7–73% between FLS)^15^, and a French FLS found that less than half of patients adhered to the 1-year follow-up course and that this was strongly associated with adherence to treatment^16^. In the same way, a Spanish FLS reported adherence of 35% to oral bisphosphonates at 12 months in patients with hip fracture^17^. On the other hand, the FLS from Seville reported treatment adherence in the first year after of 96% in both sexes^18^.

The FLS model is effective compared with standard care, although not all patients with hip fracture are candidates for treatment, since they may have serious comorbidities or a very short life expectancy. In addition, the patient or the patient’s family or primary care physician may reject initiation of AOM. Taking all these aspects into account, the most favorable expectation 1 year after hip fracture is that up to 60% of patients who survive remain in treatment. In other words, when analyzing adherence, we must consider whether the patients included comprise all discharged hip fractures or only those for whom treatment with AOM has been recommended.

Regarding the reasons for the high compliance rates related to the FLS model, a study found that patients with fragility fractures resulting from osteoporosis had greater adherence to medication. Thus, the study highlighted the key roles of FLS staff in helping patients recognize fragility fractures as a sign of underlying bone disease and encouraging adherence to care recommendations^19^. In addition, a single education session on bone health at baseline was shown to be associated with increased adherence^13^. Follow-up was associated with better adherence in the same study: at the time of telephone contact, one-third of patients required further advice to optimize their bone health^13^.

Patients who do not withdraw their medication from the pharmacy after the recommendation of the FLS represent an unresolved issue. In our previous report^20^, the reasons for the lack of initiation of treatment in the first 12 months were as follows: patient refusal to take treatment, 30%; unknown reasons, 29%; AOM not started by the primary care physician, 21%; gastrointestinal complaints, 9%; polypharmacy, 6%; and other diseases, 2%. After analyzing these results in our FLS and taking into account our efforts to achieve greater adherence to treatment after baseline visit, we began to fill the electronic prescription of the first treatment 1 week after the baseline visit (both inpatient and outpatient). This procedure is monitored by the medical coordinator after reviewing and signing the report. The patient is then contacted by telephone notifying of the prescription and reinforcing the message provided by the nurse a week before.

Poor adherence to bisphosphonates for treatment of osteoporosis increases the risk of fracture. A meta-analysis of 5 articles (234,737 patients) reported a mean PDC of 67%^3^. The authors observed a 46% higher risk of fracture (higher for clinical vertebral fractures) in non-adherent patients than in adherent patients.

We previously reported results on medium- and long-term persistence of treatment in the FLS^20,21^. The analysis presented here on hip fractures followed for at least 3 years revealed better persistence of AOM than those observed in standard clinical practice (persistence of alendronate and denosumab at 12 and 24 months in standard practice of 47–65% and 28–45%, respectively)^22^.

In a Canadian study on adherence in the context of FLS, PDC at 1 year was > 80% in 66.4% of patients who started treatment in the first 3 months^23^. Our results at 1 year were similar to those of the Canadian study, with a PDC > 80% of 68.3% (50.9% across the whole sample) for the entire follow-up. Other reports from the USA and France showed a PDC > 80% of around 60–70%^11,14^.

We found that around 60% of patients for whom treatment was indicated were adherent for at least 3 years. Considering that clinical trials with drugs for osteoporosis generally last ≥ 3 years, the percentage of patients who adhere to treatment for at least 3 years seems a relevant indicator of the effectiveness of an FLS.

There are few studies on adherence to osteoporosis treatment over 2 years, and none report on the FLS model. An observational study with a large sample showed that 25% of incident users of bisphosphonates continued taking treatment for up to 3 years and 14% for up to 5 years^24^. In the randomized SOS study, adherence at 3 years was 46%^25^, while in the SCOOP trial, adherence at 5 years was 26%^26^. Persistence in our FLS after a mean 5 years of follow-up was approximately 47%, i.e., almost twice that observed in non-FLS studies.

In the present report, we found a non-significant PDC > 80% in patients seen by the rheumatologist compared with a first prescription made in primary care (63% vs 54%). A Danish study found a similar persistence of AOM at up to 5 years in patients with osteoporosis, irrespective of whether they were treated by a specialist or primary care physician^27^.

An analysis of UK primary care prescriptions revealed the independent predictors of initiation of treatment to be female sex, not being obese, and living in the northeastern region of the country^10^. Another study from Spain showed that patients treated within 3 months of hip fracture discharge were more likely to be female, to have had previous osteoporosis treatment, to have a diagnosis of osteoporosis and rheumatoid arthritis and to use oral corticosteroids^29^. Female sex is thought to be linked to greater persistence^20^, since both physicians and patients are less likely to associate osteoporosis with male sex. In our study, previous treatment with AOM was associated with prescription and persistence to treatment. In our opinion, the association of persistence with previous bisphosphonate treatment is equally obvious, i.e., for patients who have already received these agents, FLS messaging reinforces the benefits of maintaining treatment. We found a higher rate of persistence to denosumab than for oral bisphosphonates, as reported in studies outside the FLS setting^7,28,29^.

We also report the first finding of an association between PDC and inpatient identification of fracture. This association is interesting, because it has consequences for patient management, reinforces the role of the nurse, and may increase the effectiveness of the FLS model. Organization of the FLS based on a nurse and a coordinator, as well as a strong liaison with primary care, is probably the best option for secondary prevention after hip fracture. In this context, a UK analysis showed that, compared with standard care, it is cost-effective to introduce an ortho-geriatrician- or a nurse-led FLS secondary care service for patients with hip fracture, mainly because of the effects on mortality as opposed to refracture^30^.

FLS reduces the frequency of refractures and mortality^1^. In a Dutch study, patients who were fully assessed after fracture at an FLS and were recommended bisphosphonates had substantially lower risks for both subsequent fragility fractures and mortality^31,32^. Although we observed a lower incidence of hip refracture in patients with a prescription of AOM, the sample of patients was too small to draw conclusions on mortality and refracture.

The main limitation of our study is its partially retrospective observational design. However, the number of patients analyzed was high, and the quality of the electronic prescription data from a public healthcare system was good. The results of our study cannot be generalized to all FLS models, since the baseline visit was managed mainly by nurses and because many of the prescriptions were made by primary care physicians.

In conclusion, long-term persistence of AOM after hip fracture in an FLS unit was around 60%. Eight out of 10 patients who started treatment adhered to their regimen for at least 3 years, and this adherence was higher when the baseline visit was in the inpatient setting.

## Data Availability

The dataset used and analysed during the current study is available at: https://www.dropbox.com/scl/fi/ummxue0l1ht1tqhqv34z1/dataset-Naranjo-et-al.xls?dl=0&rlkey=1lavrsqtvtr2z2nowktfs8ojk.

## Abbreviations

FLS: Fracture liaison service
AOM: Antiosteoporosis medication
DXA: Bone densitometry
PDC: Proportion of days covered

## References

[1] Kim SC, Kim MS, Sanfélix-Gimeno G, Song HJ, Liu J, Hurtado I, Peiró S, Lee J, Choi NK, Park BJ, Avorn J (2015). Use of osteoporosis medications after hospitalization for hip fracture: A cross-national study. Am. J. Med..

[2] Wu CH, Tu ST, Chang YF (2018). Fracture liaison services improve outcomes of patients with osteoporosis-related fractures: A systematic literature review and meta-analysis. Bone.

[3] Imaz I, Zegarra P, González-Enríquez J, Rubio B, Alcazar R, Amate JM (2010). Poor bisphosphonate adherence for treatment of osteoporosis increases fracture risk: Systematic review and meta-analysis. Osteoporos. Int..

[4] Naranjo A, Ojeda-Bruno S, Bilbao Cantarero A, Quevedo Abeledo JC, Henríquez-Hernández LA, Rodríguez-Lozano C (2014). Results of a model of secondary prevention for osteoporotic fracture coordinated by rheumatology and focused on the nurse and primary care physicians. Reumatol. Clin..

[5] Kanis JA, Cooper C, Rizzoli R, Abrahamsen B, Al-Daghri NM, Brandi ML, Cannata-Andia J, Cortet B, Dimai HP, Ferrari S, Hadji P, Harvey NC, Kraenzlin M, Kurth A, McCloskey E, Minisola S, Thomas T, Reginster JY, European Society for Clinical and Economic Aspects of Osteoporosis, Osteoarthritis and Musculoskeletal Diseases (ESCEO) (2017). Identification and management of patients at increased risk of osteoporotic fracture: Outcomes of an ESCEO expert consensus meeting. Osteoporos. Int..

[6] Dobre R, Niculescu DA, Petca RC, Popescu RI, Petca A, Poiană C (2021). Adherence to anti-osteoporotic treatment and clinical implications after hip fracture: A systematic review. J. Pers. Med..

[7] Kim SJ, Cho YJ, Lee DW (2021). Patients' first-year adherence to different anti-osteoporotic therapy after hip fractures. Injury.

[8] Cehic M, Lerner RG, Achten J, Griffin XL, Prieto-Alhambra D, Costa ML (2019). Prescribing and adherence to bone protection medications following hip fracture in the United Kingdom: Results from the World Hip Trauma Evaluation (WHiTE) cohort study. Bone Joint J..

[9] Registro nacional de fractura de cadera. Informe 2019. http://rnfc.es/wp-content/uploads/2021/03/Informe-Anual-RNFC-2019_digital-1.pdf. (Accessed 19 Aug 2021).

[10] Shah A, Prieto-Alhambra D, Hawley S, Delmestri A, Lippett J, Cooper C, Judge A, Javaid MK, REFReSH study team (2017). Geographic variation in secondary fracture prevention after a hip fracture during 1999–2013: A UK study. Osteoporos. Int..

[11] Scholten DJ, Bray JK, Wang KY, Lake AF, Emory CL (2020). Implementation of a fracture liaison service and its effects on osteoporosis treatment adherence and secondary fracture at a tertiary care academic health system. Arch. Osteoporos..

[12] Chandran M, Cheen M, Ying H, Lau TC, Tan M (2016). Dropping the ball and falling off the care wagon. Factors correlating with nonadherence to secondary fracture prevention programs. J. Clin. Densitom..

[13] Hui N, Fraser S, Wong PKK (2020). Patients discharged from a fracture liaison service still require follow-up and bone health advice. Arch. Osteoporos..

[14] Delbar A, Pflimlin A, Delabrière I, Ternynck C, Chantelot C, Puisieux F, Cortet B, Paccou J (2020). Persistence with osteoporosis treatment in patients from the Lille University Hospital Fracture Liaison Service. Bone.

[15] Fracture Liaison Service Database (FLS-DB). Royal College of Physicians: https://www.rcplondon.ac.uk/projects/outputs/fls-database-annual-report-2020. (Accessed 20 July 2021).

[16] Mugnier B, Daumas A, Doddoli S (2020). Adherence to fracture liaison service programs in patients over 70: The hidden part of the iceberg. Osteoporos. Int..

[17] Gamboa A, Duaso E, Marimón P, Sandiumenge M, Escalante E, Lumbreras C, Tarrida A (2018). Oral bisphosphonate prescription and non-adherence at 12 months in patients with hip fractures treated in an acute geriatric unit. Osteoporos. Int..

[18] Olmo-Montes FJ, Hernández-Cruz B, Miranda MJ, Jimenez-Moreno MD, Vázquez-Gámez MÁ, Giner M, Colmenero MA, Pérez-Venegas JJ, Montoya-García MJ (2021). The fracture liaison service of the Virgen Macarena University Hospital reduces the gap in the management of osteoporosis, particularly in men. It meets the international osteoporosis foundation quality standards. J. Clin. Med..

[19] Luc M, Corriveau H, Boire G, Filiatrault J, Beaulieu MC, Gaboury I (2018). Patient-related factors associated with adherence to recommendations made by a fracture liaison service: A mixed-method prospective study. Int. J. Environ. Res. Public Health..

[20] Naranjo A, Ojeda-Bruno S, Bilbao-Cantarero A, Quevedo-Abeledo JC, Diaz-González BV, Rodríguez-Lozano C (2015). Two-year adherence to treatment and associated factors in a fracture liaison service in Spain. Osteoporos. Int..

[21] Naranjo A, Fernández-Conde S, Ojeda S (2017). Preventing future fractures: Effectiveness of an orthogeriatric fracture liaison service compared to an outpatient fracture liaison service and the standard management in patients with hip fracture. Arch. Osteoporos..

[22] Reyes C, Tebe C, Martinez-Laguna D (2017). One and two-year persistence with different anti-osteoporosis medications: A retrospective cohort study. Osteoporos. Int..

[23] Senay A, Fernandes JC, Delisle J, Morin SN, Perreault S (2019). Persistence and compliance to osteoporosis therapy in a fracture liaison service: A prospective cohort study. Arch. Osteoporos..

[24] Friesen KJ, Bugden S, Falk J (2020). Time to benefit and the long-term persistence of new users of oral bisphosphonates. J. Bone Miner. Metab..

[25] Merlijn T, Swart KM, van Schoor NM (2019). The effect of a screening and treatment program for the prevention of fractures in older women: A randomized pragmatic trial. J. Bone Miner. Res..

[26] Shepstone L, Lenaghan E, Cooper C (2018). Screening in the community to reduce fractures in older women (SCOOP): A randomised controlled trial. Lancet.

[27] Hitz MF, Arup S, Holm JP, Soerensen AL, Gerds TA, Jensen JB (2020). Outcome of osteoporosis evaluation, treatment, and follow-up in patients referred to a specialized outpatient clinic compared to patients in care of general practitioners. Arch. Osteoporos..

[28] Fahrleitner-Pammer A, Papaioannou N, Gielen E, FeudjoTepie M, Toffis C, Frieling I, Geusens P, Makras P, Boschitsch E, Callens J, Anastasilakis AD, Niedhart C, Resch H, Kalouche-Khalil L, Hadji P (2017). Factors associated with high 24-month persistence with denosumab: Results of a real-world, non-interventional study of women with postmenopausal osteoporosis in Germany, Austria, Greece, and Belgium. Arch. Osteoporos..

[29] Hurtado I, García-Sempere A, Peiró S, Rodríguez-Bernal C, Sanfélix-Genovés J, Sanfélix-Gimeno G (2020). Trends and geographical variability in osteoporosis treatment after hip fracture: A multilevel analysis of 30,965 patients in the region of Valencia, Spain. J. Bone Miner. Res..

[30] Leal J, Gray AM, Hawley S, Prieto-Alhambra D, Delmestri A, Arden NK, Cooper C, Javaid MK, Judge A, TheREFReSH Study Group (2017). Cost-effectiveness of orthogeriatric and fracture liaison service models of care for hip fracture patients: A population-based study. J. Bone Miner. Res..

[31] Huntjens KM, van Geel TA, van den Bergh JP (2014). Fracture liaison service: Impact on subsequent nonvertebral fracture incidence and mortality. J. Bone Joint Surg. Am..

[32] van Geel TACM, Bliuc D, Geusens PPM, Center JR, Dinant GJ, Tran T, van den Bergh JPW, McLellan AR, Eisman JA (2018). Reduced mortality and subsequent fracture risk associated with oral bisphosphonate recommendation in a fracture liaison service setting: A prospective cohort study. PLoS ONE.

